# Carbon-Material-Modified Polyester Nonwoven Composites with Enhanced Mechanical, Electrical, and Thermal Properties

**DOI:** 10.3390/polym18141718

**Published:** 2026-07-13

**Authors:** Wenyan Gu, Xinyi Jin, Jiaqiao Zhang, Nannan Guo, Yu Shi, Jiang Shi, Xiangrong Lan, Licheng Zhu

**Affiliations:** 1 School of Textile and Clothing, Nantong University, Nantong 226019, China; 2 School of Mechanical Engineering, Southeast University, Nanjing 211189, China; 3 National Local Joint Laboratory for Advanced Textile Processing and Clean Production, Wuhan Textile University, Wuhan 430200, China

**Keywords:** graphene, nonwoven fabric, polyester, polyurethane, functional composite, smart textile, protective clothing, thermal management, electrical conductivity

## Abstract

Carbon nanotube (CNT)- and graphene flake (GF)-modified polyester (PET) nonwoven composites were prepared using a one-sided impregnation process with waterborne polyurethane (PU) as the binder. The objective of this work was to clarify how the geometry and loading of one-dimensional CNTs and two-dimensional GFs regulate conductive network formation, anisotropic mechanical behavior, and thermal response in PU/PET nonwoven composites. The novelty of the study lies in the direct comparison of CNT and GF fillers in the same nonwoven/PU matrix and in correlating filler morphology with mechanical reinforcement, electrical conductivity, and textile-related thermal management performance. The sample codes C5 and C6 represent CNT contents of 5 and 6 wt.%, respectively, while G4 and G6 represent GF contents of 4 and 6 wt.%, respectively. Scanning electron microscopy (SEM) showed that GF tended to form sheet-like coatings on fiber surfaces and to fill inter-fiber pores, whereas CNTs showed more local aggregation because of their high surface energy. The composites exhibited anisotropic tensile behavior, with higher tensile strength in the longitudinal direction than in the transverse direction. In the longitudinal tensile test, G4 reached a tensile strength of 13.01 MPa, while C5 reached 11.35 MPa. With increasing carbon material content, both the electrical and thermal conductivities of the composites increased. The electrical conductivity reached 0.02100 S/cm for C6 and 0.05893 S/cm for G6. The thermal conductivity of the CNT/PU/PET composites increased from 0.1163 to 0.1923 W/(m·K), whereas that of the GF/PU/PET composites increased from 0.1793 to 0.2537 W/(m·K). Infrared thermal imaging further indicated that carbon material addition produced faster heating and slower heat dissipation than the unmodified PU/PET sample. These results provide a useful reference for developing multifunctional nonwoven composites for smart textiles, special protective clothing, wearable thermal management layers, and flexible electronic textile substrates.

## 1. Introduction

Functional textiles for apparel and protective equipment are evolving from passive covering materials into multifunctional platforms that integrate mechanical reliability, thermal regulation, electrical response, electromagnetic protection, and wearable comfort. In particular, garments for special equipment personnel, emergency rescue, outdoor operation, and other demanding service scenarios require lightweight and flexible materials that can provide thermal comfort, antistatic performance, electromagnetic attenuation, and compatibility with wearable electronic devices. Previous studies by Gu et al. have demonstrated the importance of nonwoven structural design for clothing thermal insulation and air permeability, as well as the feasibility of polyurethane/polyester (PU/PET) nonwoven composites for flexible microwave-absorbing and electromagnetic functional materials [1,2,3,4,5]. These results suggest that PET nonwovens can serve as processable fibrous skeletons for advanced apparel-related functional composites when combined with appropriate conductive fillers and polymer binders.

Nonwoven fabrics are widely used in textile and apparel materials because of their porous structure, low cost, short processing route, light weight, flexibility, and high structural designability [6,7,8,9,10,11,12,13]. Compared with conventional woven or knitted fabrics, nonwovens can be readily tailored in fiber orientation, thickness, pore distribution, and layered structure, making them suitable for clothing interlayers, protective liners, thermal regulation components, and flexible substrates for wearable devices. However, conventional PET nonwovens are intrinsically insulating and generally show limited thermal-conduction capacity and insufficient mechanical reinforcement for high-performance functional clothing systems. These limitations restrict their direct use in smart protective garments, active/passive thermal management layers, antistatic or electromagnetic-protective linings, and textile-based sensing substrates.

Nanomaterial modification is an effective strategy for converting ordinary nonwovens into multifunctional textile composites. Among various functional fillers, carbon nanotubes (CNTs) and graphene flakes (GFs) are especially attractive because of their high aspect ratio or two-dimensional sheet structure, high mechanical strength, excellent electrical and thermal conductivities, and chemical stability [14,15,16,17,18,19]. When introduced into a fibrous nonwoven matrix, CNTs and GFs can form electrically conductive pathways through direct filler–filler contact, tunneling between neighboring particles separated by thin polymer layers, and progressive percolation as the filler content increases. Under deformation, changes in contact number, tunneling distance, and local network continuity can alter resistance, which is the basis for damage-sensing and structural health monitoring concepts reported for CNT- and graphene-modified joint systems [20,21]. In a PET nonwoven/PU system, these network mechanisms are further affected by fiber orientation, inter-fiber pores, filler dispersion, interfacial adhesion with the binder, and the preservation of the original porous fabric structure.

For apparel-oriented functional composites, property enhancement must be balanced with flexibility, porosity, durability, and wearing comfort. High electrical conductivity is beneficial for antistatic protection, electromagnetic wave management, wearable sensing, and electronic textile integration, while enhanced thermal conductivity and thermal stability can support heat dissipation, localized warming, thermal buffering, and environmental protection in special clothing systems [22,23,24,25,26,27,28,29,30]. However, excessive filler loading may induce CNT or GF aggregation, reduce the uniformity of the conductive network, block pores, increase local stiffness, and weaken stress transfer in the composite. Therefore, it is necessary to clarify how the type and content of carbon materials regulate the microstructure and macroscopic properties of PET nonwoven composites.

In this study, CNT- and GF-modified PET nonwoven composites were fabricated by a one-sided impregnation method using waterborne PU as the binder. The present work was motivated by three practical challenges in carbon material textile composites: maintaining filler dispersion while increasing filler loading, constructing continuous conductive and thermally conductive networks without severely blocking the porous nonwoven structure, and balancing mechanical reinforcement with flexibility and textile-related thermal performance. The effects of carbon material type and content on microstructure, anisotropic tensile properties, in-plane electrical conductivity, thermal conductivity, infrared thermal behavior, and thermal stability were investigated. The enhancement mechanisms were discussed in terms of filler morphology, fiber surface coating, conductive network construction, and physical interlocking with the PU/PET matrix. This work aims to provide theoretical and experimental support for the development of multifunctional PET nonwoven composites for smart textiles and special protective apparel, including thermal management interlayers, conductive/antistatic layers, electromagnetic-protective liners, and flexible substrates for wearable sensing.

## 2. Materials and Methods

### 2.1. Materials

CNT slurry with a solid content of 15 wt.% and GF slurry with a solid content of 18 wt.% were purchased from Nakaite New Materials Technology Co., Ltd., Suqian, China. According to SEM observations reported in our previous study using the same commercial carbon material slurries, the CNT diameter was approximately 74.1 nm and the lateral size of the GF was approximately 2.4 μm [4]. The average CNT length was not provided by the supplier and was therefore not reported in this work. Waterborne polyurethane (PU, YC-601C, solid content 43 wt.%) and thickener (YC-100B) were supplied by Yuancheng New Materials Technology Co., Ltd., Hefei, China. Dispersant (6508) and defoamer (DF904A) were provided by Guangzhou Aohe New Materials Co., Ltd., Guangzhou, China. Polyester (PET) needle-punched nonwoven fabric with a thickness of approximately 1 mm was purchased from Yiwu Duandi E-Commerce Co., Ltd., Yiwu, China. Deionized water was prepared in the laboratory.

### 2.2. Preparation of Composite Samples

**Figure 1:**
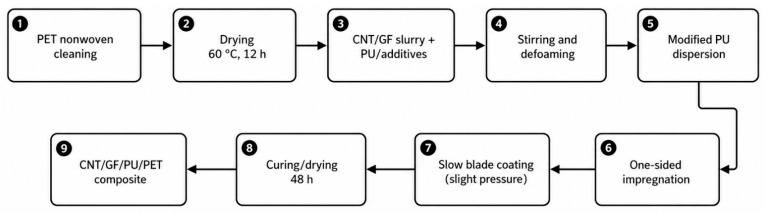
Schematic illustration of the one-sided impregnation process used to prepare CNT/GF-modified PU/PET nonwoven composites.

CNT/PU/PET composite samples were prepared by a one-sided impregnation process, as shown in Figure 1. First, the PET nonwoven substrate was cleaned with deionized water, dried at 60 °C for 12 h, and cooled naturally. CNT slurry with different mass fractions was then mixed with PU, dispersant, defoamer, thickener, and deionized water, followed by mechanical stirring and defoaming to obtain a relatively homogeneous modified PU dispersion. The dispersion was poured into the bottom of a mold to form a uniform liquid layer. The PET nonwoven fabric was placed onto the liquid layer and gently pressed to ensure full contact and impregnation through the thickness. The side in direct contact with the impregnation solution was defined as the backside, and the opposite side was defined as the frontside. For GF/PU/PET samples, the same process was used, except that slow blade coating under slight pressure was applied when necessary because the GF-containing dispersion showed reduced flowability at higher GF contents. The impregnated samples were naturally dried for 48 h and then removed from the mold. Seven CNT/PU/PET composite samples with different CNT contents were prepared and denoted as C0-C6, as shown in Table 1. Graphene-modified PET nonwoven composites were prepared using the same procedure and denoted as G0–G6.

### 2.3. Characterization

The surface morphologies of the PET nonwoven fabric, CNT/PU/PET composites, and GF/PU/PET composites were observed using field-emission scanning electron microscopy (FE-SEM, Gemini 300, Carl Zeiss, Germany). During SEM observation, an accelerating voltage of 5 kV was used, and the samples were sputter-coated with gold to improve surface conductivity. The mechanical properties of the composites were evaluated using an Instron 5969 universal testing machine. Tensile strength, elongation at break, Young’s modulus, and stress–strain curves were obtained. The test was performed according to ISO 37:2005 [31]. Samples were cut into dumbbell-shaped specimens with dimensions of 35 mm × 6 mm. The gauge length was set to 12 mm, and the crosshead speed was 200 mm/min. To ensure the reliability of the results, each sample was tested three times in both the longitudinal and transverse directions. The electrical properties of the composites were measured at room temperature using a four-point probe method with an ST2722 powder resistivity tester (Suzhou Jingge Electronics Co., Ltd., Suzhou, China). Samples were placed in a polytetrafluoroethylene sample holder with an inner diameter of 15 mm. A plunger equipped with a four-point probe was pressed onto the sample by a hydraulic system under pressures ranging from 0 to 18 MPa. The resistivity values under different pressures were automatically recorded by the control software. For infrared (IR) thermal imaging, samples with dimensions of 50 mm × 40 mm were vertically fixed at the bottom of a Galanz microwave oven, model P70D20P-N9(W0) (Galanz, Foshan, China), without a rotating plate. Microwave heating was initiated at the 10th second and terminated at the 14th second, at which point the furnace door was opened. An FLIR ONE PRO infrared thermal imager (Teledyne FLIR Commercial Systems, Wilsonville, OR, USA) was used to record the entire heating and cooling process, and representative thermal images were extracted from the recorded videos. Thermal conductivity was measured according to ASTM D5470 [32] using a DRL-III thermal conductivity tester (Xiangtan Xiangyi Instrument Co., Ltd., Xiangtan, China). Circular specimens with a diameter of 30 mm were used. During the test, the hot-side temperature was set to 25 °C, and the cold-side temperature was set to 20 °C. Thermogravimetric analysis (TG) was performed using a NETZSCH STA 449 F5 simultaneous thermal analyzer (Netzsch, Selb, Germany). The samples were heated from 30 to 600 °C at a heating rate of 10 °C/min. The derivative thermogravimetry (DTG) curves were obtained from the corresponding TG curves. The thickness of the composites was measured using a YG141D digital fabric (Changzhou No.1 Textile Equipment Co., Ltd., Changzhou, China) thickness tester according to GB/T 3820-1997 [33]. Five positions were randomly selected for each sample, and the average value was calculated. The mass per unit area was determined according to ISO 9073-1:2023 [34], MOD, using a YP 20002 digital electronic balance (Shanghai Yueping Scientific Instrument Company, Shanghai, China). Data processing was performed using ORIGIN (v2024).

## 3. Results and Discussion

### 3.1. Morphological Analysis

**Figure 2:**
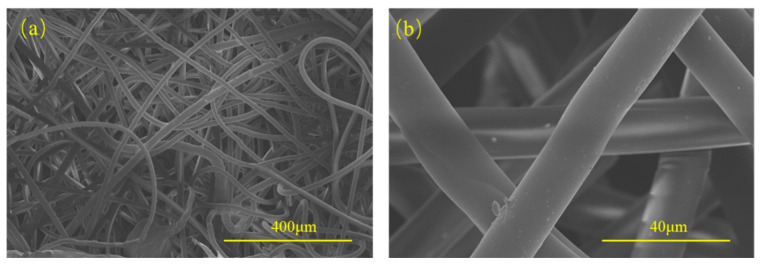
SEM images of PET nonwoven fabric: (**a**) low-magnification image; (**b**) high-magnification image.

The microstructures of PET, CNT/PU/PET, and GF/PU/PET samples were investigated by SEM. The SEM images of the PET substrate are shown in Figure 2.

As shown in Figure 2a, the SEM image of the PET nonwoven fabric shows a randomly interlaced and entangled fibrous structure, forming a relatively loose porous network with abundant internal pores. The magnified SEM image in Figure 2b further reveals that the PET fiber surfaces are not completely smooth. Several barbs and protrusions can be observed, which may be attributed to the repeated penetration and movement of needles during the needle-punching process.

**Figure 3:**
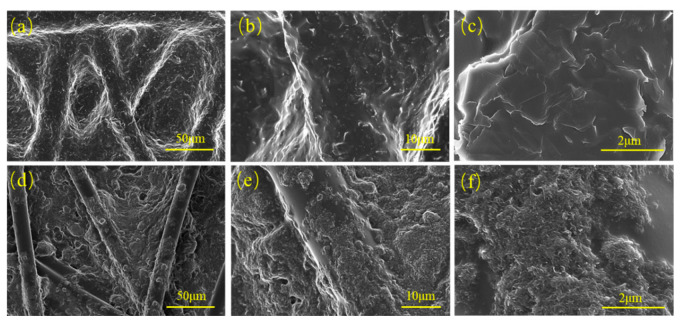
SEM images of GF/PU/PET and CNT/PU/PET: (**a**) G4 at 500×; (**b**) G4 at 3000×; (**c**) G4 at 10,000×; (**d**) C6 at 500×; (**e**) C6 at 3000×; (**f**) C6 at 10,000×.

Figure 3a presents the overall morphology of the GF/PU/PET sample. The PET fibers are extensively coated by the impregnating solution, which also fills the gaps among fibers. G4 was selected for SEM observation because it showed the best longitudinal tensile performance among the GF/PU/PET samples, whereas C6 was selected to show the morphology of the CNT system at high filler loading and at the highest electrical conductivity. Thus, the SEM comparison was intended to reveal representative morphology–property relationships rather than to compare two samples at identical filler contents. As shown in Figure 3b, several particles are distributed on the surface of GF/PU/PET, which can be attributed to GF sheets physically adhering to the fiber surfaces. Figure 3c clearly reveals the sheet-like morphology of GF, with lateral dimensions of approximately 2–3 μm. The two-dimensional sheet structure of GF provides a larger contact area with the PU-coated fibers than the one-dimensional CNTs, and it can bridge neighboring fibers and pores more easily. This morphology favors stress transfer and conductive path formation. Figure 3d–f show the SEM images of CNT/PU/PET. The PET fibers are also coated with the impregnating solution; however, the coating is less uniform than that of the G4 sample, and a few uncovered regions can still be observed. Moreover, the magnified images reveal that although CNTs adhere to the fiber surfaces, CNT aggregation still occurs. This is mainly related to the high aspect ratio, large specific surface area, and strong van der Waals attraction of CNTs, which increase their tendency to entangle and form local bundles. The aggregation may cause local heterogeneity in composite properties and consequently reduce the strength and stability of the material. Because FTIR and XPS analyses were not conducted in this work, the above discussion is limited to morphology-based evidence and macroscopic property changes, and no conclusion is made regarding chemical bonding at the filler/PU/PET interfaces.

### 3.2. Mechanical Properties

**Figure 4:**
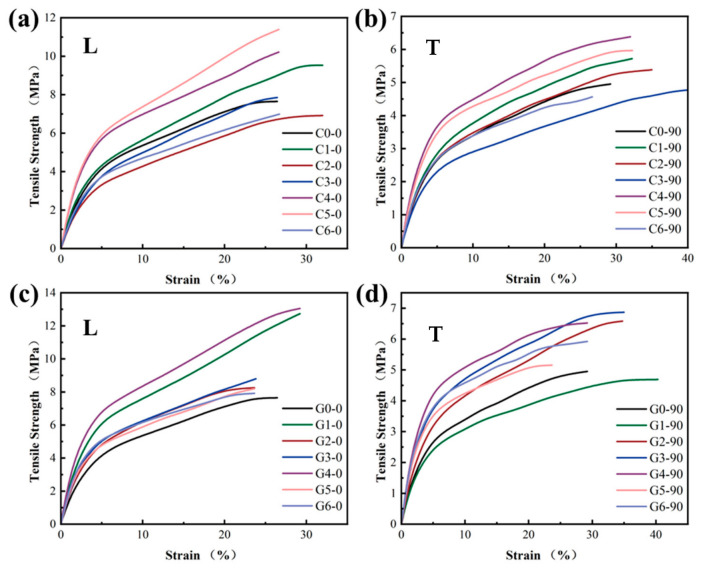
Stress–strain curves of CNT/PU/PET and GF/PU/PET composites: (**a**) longitudinal direction of CNT/PU/PET; (**b**) transverse direction of CNT/PU/PET; (**c**) longitudinal direction of GF/PU/PET; (**d**) transverse direction of GF/PU/PET.

**Figure 5:**
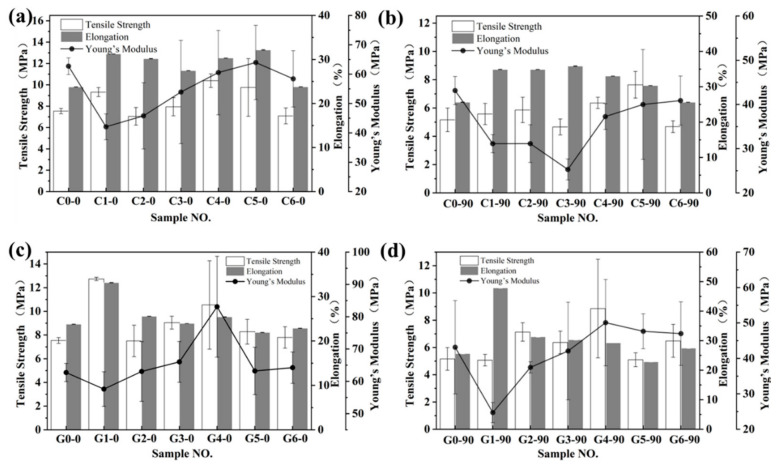
Elongation at break, tensile strength, and Young’s modulus of composites with different carbon material contents: (**a**) longitudinal direction of CNT/PU/PET; (**b**) transverse direction of CNT/PU/PET; (**c**) longitudinal direction of GF/PU/PET; (**d**) transverse direction of GF/PU/PET.

The mechanical properties of the composites were evaluated using tensile tests, and the corresponding stress–strain curves are shown in Figure 4, where (a) and (c) are for the longitudinal direction (L), and b and d for the transverse direction (T). The tensile strength, elongation at break, and Young’s modulus values are summarized in Figure 5.

As can be seen from the figures, the composites exhibited obvious anisotropic mechanical behavior in the transverse and longitudinal directions. The tensile properties in the longitudinal direction were significantly superior to those in the transverse direction. This phenomenon is closely associated with the manufacturing process of the PET nonwovens. In the fabrication of PET nonwovens, the direction of fiber carding and entanglement is typically aligned with the machine direction, which leads to denser fiber entanglement along this orientation and consequently results in superior longitudinal tensile properties. Furthermore, the advantage of longitudinal mechanical properties enables the composite to better withstand tensile stresses along the fiber direction in service, thereby improving the reliability and stability of the material during service. Consequently, after the PET nonwovens were coated with the impregnation solution, the overall tensile properties of the resulting composites were improved. As shown in Figure 4, the tensile properties of the composites increased with increasing carbon material mass ratio within an appropriate range. However, once the carbon material mass ratio reached a certain level, the tensile properties exhibited a declining trend. In the longitudinal tensile tests, C5 achieved a maximum tensile stress of 11.35 MPa, while G4 reached 13.01 MPa. With further increases in the carbon material mass fraction, the tensile stress of C6 dropped to 6.97 MPa, and those of G5 and G6 decreased to 8.17 MPa and 7.89 MPa, respectively. A similar trend was observed in the transverse tensile properties. As can be seen from Figure 5, G4 exhibited the highest longitudinal breaking strength and elongation at break, which were 13.01 MPa and 29.2%, respectively, indicating that an appropriate amount of carbon material can improve the toughness and strength of the composite. For the CNT/PU/PET samples in Figure 5a), the decrease from C1-0 to C2-0 and the subsequent increase from C2-0 to C4-0 can be explained by the competition between local CNT dispersion heterogeneity and filler-induced reinforcement. At low CNT contents, a small change in local CNT distribution may disturb the continuity of the PU coating and create weak points, so a slight decrease can occur from C1-0 to C2-0. When the CNT content further increases to C3-0 and C4-0, more CNTs participate in fiber bridging and stress transfer, leading to improved tensile strength. If the mass ratio of carbon material is excessively high, the contact probability among carbon materials increases significantly, providing more collision opportunities and aggregation pathways for CNTs and GF, which are thermodynamically prone to agglomeration. Driven by attractive forces such as hydrophobic interactions and van der Waals attraction, they approach one another and become agglomerated. When these agglomerates transfer stress in the composite, the agglomerated regions tend to form stress concentrations, eventually leading to a decline in mechanical properties.

### 3.3. Electrical Conductivity

**Figure 6:**
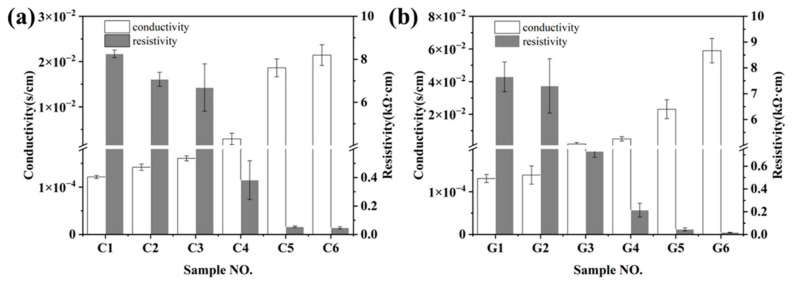
Electrical conductivity of the composites: (**a**) CNT/PU/PET; (**b**) GF/PU/PET.

Carbon materials have good intrinsic electrical conductivity. In the CNT/PU/PET and GF/PU/PET composites, the measured conductivity is governed not only by the intrinsic conductivity of the fillers but also by the formation of filler–filler contacts, tunneling between adjacent fillers, and the percolated conductive network along the coated fiber surfaces and inter-fiber pores. Therefore, the four-point probe method was used to test the electrical conductivity of the composites, and the results are shown in Figure 6.

As can be seen from Figure 6a, the electrical conductivity of the CNT/PU/PET composites gradually increases with an increase in the CNT mass fraction, while the resistivity decreases accordingly. The conductivities of samples C1 to C3 are all close to zero, indicating that an effective conductive network has not yet been formed at these CNT mass fractions. As the CNT mass fraction continues to increase, the conductivities of C4 to C6 begin to rise rapidly. At this stage, CNTs gradually form continuous conductive pathways, thereby significantly improving the electrical performance of the material. Sample C6 exhibits the highest conductivity, reaching 0.02100 S/cm. From Figure 6b, it is observed that with the progressive increase in GF mass ratio, the electrical conductivity of the GF/PU/PET composites continuously increases, while the resistivity decreases subsequently. G1 shows a relatively low conductivity of 1.31 × 10^−4^ S/cm, while the resistivity of G1 is high, which is 7.65 kΩ·cm, indicating that the conductivity of G1 is poor and the movement of electrons in the material is difficult. As the GF mass ratio increases, the conductivity of G3 rises to 0.00173 S/cm, and the resistivity drops to 0.7297 kΩ·cm, indicating improved electrical properties. When it continues to rise to G6, the conductivity increases significantly to 0.05893 S/cm, the resistivity decreases to 0.01713 kΩ·cm, and the conductivity of the material is significantly enhanced. At the same filler mass fraction, the GF/PU/PET samples generally show higher conductivity than the corresponding CNT/PU/PET samples, suggesting that GF is more efficient in constructing an in-plane conductive network in this nonwoven structure. From the viewpoint of structural health monitoring, the higher baseline conductivity and more continuous sheet-like network of GF/PU/PET may provide a more favorable electrical pathway for resistance-based sensing. However, actual damage sensitivity still needs to be verified under coupled mechanical loading and resistance monitoring.

### 3.4. Thermal Properties

**Figure 7:**
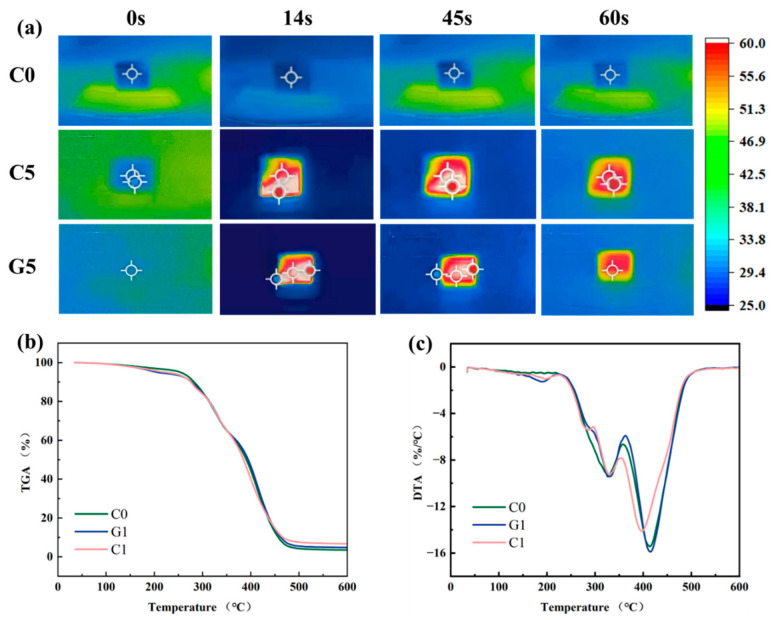
Thermal behavior of the composites: (**a**) IR thermal images of C0, C5, and G5 at different times; (**b**) thermogravimetric analysis (TG) curves of C0, C1, and G1; (**c**) derivative thermogravimetry (DTG) curves of C0, C1, and G1.

The thermal behavior of C0, CNT/PU/PET, and GF/PU/PET composites was evaluated by IR thermal imaging, TG, and thermal conductivity measurement, as shown in Figure 7. For TG and DTG analysis, C0, C1, and G1 were selected as representative samples to compare the unfilled PU/PET system with the initial addition of CNT or GF under the same testing conditions. Samples with higher filler contents were not included in the TG/DTG comparison; therefore, the TG/DTG results should be interpreted as representative thermal stability data rather than a complete filler-loading-dependent thermal degradation analysis.

Figure 7a presents the IR thermal images of the surfaces of samples at different heating times. In the IR thermal images, the lowest, highest, and target object temperatures can be displayed simultaneously, and the corresponding positions are all encircled in white. The temperature of the pure impregnated PU sample (i.e., C0 or G0) remained relatively stable, essentially within the range of 25.4–26.0 °C, indicating weak microwave heating or rapid heat dissipation and consequently poor heat retention. This suggests that the C0 undergoes negligible temperature change under microwave heating, reflecting its low thermal conductivity. In contrast, after the heating was stopped at 14 s, the temperatures of the C5 and G5 samples did not decrease significantly. At 45 s, the thermal diffusion area displayed in the thermal images expanded. By 60 s, the diffusion area shrank and the thermal intensity declined, demonstrating a fast heating and slow dissipation characteristic. This indicates that the incorporation of carbon materials substantially improves the thermal stability of the composites.

Figure 7b,c presents the TG and DTG curves of the representative composites. The TG curve reflects the mass change of the materials during heating. Typically, the thermal stability of a material can be evaluated by its residual mass at a specific temperature. Throughout the heating process, C0 exhibits a lower residual mass, indicating that the PU/PET composite without carbon filler has poorer thermal stability at high temperatures and is more prone to decomposition. In contrast, G1 and C1 show relatively better thermal stability. Figure 7c displays the DTG curves of the composites. The curves of C0, C1, and G1 exhibit similar characteristics. As the temperature increases, the maximum decomposition rate peaks at approximately 400 °C for all samples, indicating significant mass loss at this temperature. Upon further heating, the DTG curves gradually level off, suggesting a progressive deceleration of the decomposition process. Because only representative low-filler samples were tested by TG/DTG, the influence of filler loading on thermal degradation should be examined in future work.

**Figure 8:**
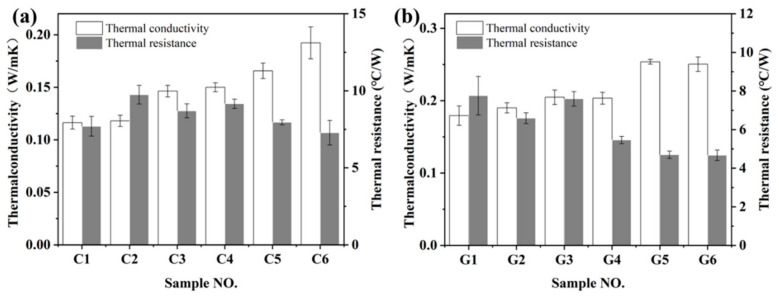
Thermal conductivity and thermal resistance of the composites: (**a**) CNT/PU/PET; (**b**) GF/PU/PET.

To investigate the thermal conductivity of the composites after the incorporation of carbon materials, the thermal conductivity and thermal resistance of the CNT/PU/PET and GF/PU/PET composites were further measured, and the results are shown in Figure 8.

As shown in Figure 8a, the thermal conductivity of the CNT/PU/PET composites generally increases with increasing CNT mass ratio. The thermal conductivity of C1 is 0.1163 W/(m·K), and that of C6 reaches 0.1923 W/(m·K) when the CNT mass ratio increases to 6 wt.%, indicating that the incorporation of CNTs effectively enhances the heat conduction capability of the CNT/PU/PET composites. With the mass ratio range from C1 to C3, the thermal conductivity of the composites increases relatively rapidly, from 0.1163 W/(m·K) to 0.1463 W/(m·K). From C4 to C6, the increase in conductivity becomes slower, which may be attributed to the agglomeration of CNTs. Due to their large specific surface area and high surface energy, CNTs tend to attract each other and agglomerate when their mass ratio is too high. This agglomeration can impair the uniformity of thermal conduction in the graded structure and increase the thermal resistance along the heat transfer pathways, thereby hindering further improvement of the thermal conductivity. Thermal resistance is inversely proportional to thermal conductivity; when thermal conductivity increases, thermal resistance gradually decreases. Taking C1 and C6 as examples, the thermal resistance of C1 is 7.6920 °C/W, while that of C6 is 7.2744 °C/W. Figure 8b reveals that the thermal conductivity behavior of the GF/PU/PET composites follows a similar trend to that of the CNT/PU/PET composites. As the GF mass ratio gradually increases from G1 to G6, the thermal conductivity shows an overall upward trend. The thermal conductivity of G1 is 0.1793 W/(m·K), and with increasing GF mass ratio, it reaches a maximum value of 0.2537 W/(m·K) at G5. However, when the mass ratio of GF reaches 5 wt.% (G5), the effect of increasing the mass fraction of GF on improving the thermal conductivity is no longer obvious. In terms of thermal resistance, the change trend is opposite to that of thermal conductivity. The thermal resistance of G1 is 7.7601 °C/W. With an increase in the mass fraction of GF, the thermal resistance gradually decreases until it drops to 4.6667 °C/W at G6, indicating that the thermal conductivity of GF/PU/PET composites is enhanced, while the resistance of heat transfer is reduced. This shows that the higher the thermal conductivity of the material, the smaller the resistance encountered when heat passes through the material and the easier it is to conduct heat, which is in line with the basic principle of heat conduction.

## 4. Conclusions

In this study, a series of CNT- and GF-modified PET nonwoven functional composites were successfully prepared by an impregnation method for potential use in smart textiles and special protective apparel. Through systematic characterization of the microstructure and macroscopic properties, the following main conclusions were obtained:

(1) GF more uniformly coated and filled the nonwoven structure, whereas CNTs tended to agglomerate. After modification with carbon materials, the composites maintained the fibrous porous structure of the PET nonwoven composites and exhibited the structural integrity and mechanical strength required for flexible textile interlayers and protective components. The longitudinal tensile strength reached 13.01 MPa for G4 and 11.35 MPa for C5, indicating that appropriate carbon material loading can improve the strength and toughness of the composites. The discussion of filler/PU/PET interfacial behavior is based on SEM morphology and macroscopic property changes because FTIR and XPS analyses were not conducted.

(2) The in-plane electrical conductivity of the composites increased significantly with increasing carbon material content. GF showed greater advantages than CNTs in constructing conductive networks. For example, the conductivity of G6 reached 0.05893 S/cm, while that of C6 was 0.02100 S/cm. This conductive behavior is beneficial for developing PET nonwoven composites with antistatic, electromagnetic wave attenuation, wearable sensing, and electronic-textile integration potential.

(3) The introduction of carbon materials markedly improved the thermal conductivity of the composites and produced a thermal response characterized by rapid heating and slow heat dissipation. These properties are useful for clothing-related thermal management, especially for functional interlayers designed for heat diffusion, localized warming, and protection in cold, outdoor, or special working environments.

In conclusion, this study confirms the technical feasibility of preparing multifunctional textile composites by modifying low-cost PET nonwovens with carbon materials. In terms of in-plane electrical conduction and heat conduction performance, GF/PU/PET composites show more prominent advantages than CNT/PU/PET composites, indicating their potential for smart textiles, special equipment personnel clothing, antistatic and electromagnetic-protective liners, wearable thermal management layers, and flexible sensing substrates. Future research should further optimize air and moisture permeability, softness, washing durability, flame retardancy, skin contact safety, electromagnetic shielding performance, coupled mechanical–electrical damage sensing, and garment system performance to verify their practical applicability in textile and apparel products.

## Figures and Tables

**Table 1:** Sample codes and carbon material contents of the composites.

Sample Code	CNT Content/wt.%	Sample Code	GF Content/wt.%
C0	0	G0	0
C1	1	G1	1
C2	2	G2	2
C3	3	G3	3
C4	4	G4	4
C5	5	G5	5
C6	6	G6	6

## Data Availability

The original contributions presented in this study are included in the article. Further inquiries can be directed to the corresponding authors.
